# Meet the authors: Yong Chen, Xiqun (Michael) Chen, and Ziyou Gao

**DOI:** 10.1016/j.patter.2023.100877

**Published:** 2023-11-10

**Authors:** Yong Chen, Xiqun (Michael) Chen, Ziyou Gao

**Affiliations:** 1 Institute of Intelligent Transportation Systems, College of Civil Engineering and Architecture, Zhejiang University, Hangzhou 310058, China; 2 Zhejiang University/University of Illinois Urbana-Champaign (ZJU-UIUC) Institute, Haining 314400, China; 3 School of Systems Science, Beijing Jiaotong University, Beijing 100044, China

## Abstract

In their recent publication in *Patterns*, the authors proposed a novel multi-scale unified mobility model to capture the universal-scale laws of individual and population movement within urban agglomerations. This People of Data highlights the contributions of their work to the field and the critical role data science plays in research and the research community.

## Main text

### What would you like to share about your background?

**Figure:**
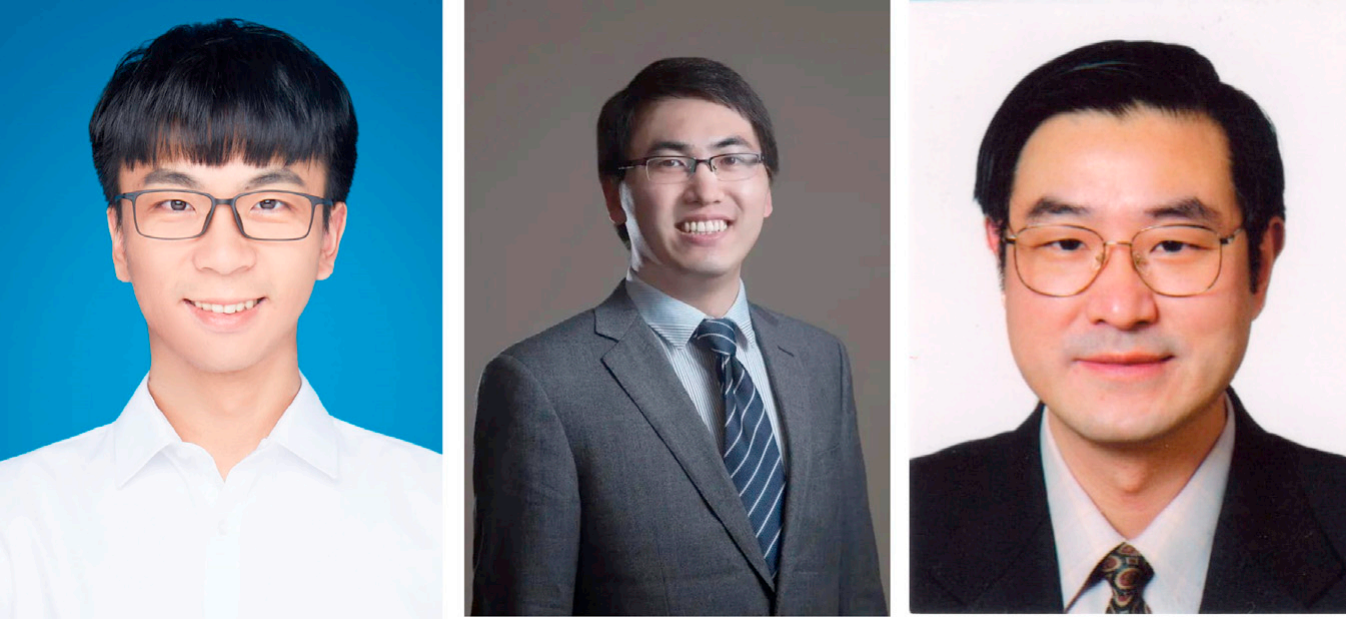
Figure: From left to right: Yong Chen, Xiqun (Michael) Chen, and Ziyou Gao

**Yong Chen**: I’m pursuing a PhD in road and traffic engineering at Zhejiang University, China. My undergraduate and master’s degrees are computer science and technology and management statistics, respectively. Drawing upon my interdisciplinary background, I focus on modeling research based on artificial intelligence and statistical theory. Prior to my PhD studies, my research focused on AI-based time series forecasting. In 2021, I joined the research group led by Professor Xiqun (Michael) Chen and embarked on research related to human mobility modeling. I am deeply grateful to my supervisor for guiding me toward this cutting-edge research topic. Studying human travel behavior and patterns appears to be more closely aligned with real life and presents greater challenges compared to my previous research endeavors.

**Xiqun (Michael) Chen:** In 2004, I was lucky to begin my undergraduate study at the Department of Civil Engineering, Tsinghua University, Beijing, China. With curiosity about urban traffic signal control and transportation planning, I pursued my PhD under the supervision of Professor Qixin Shi and co-supervision of Professor Li Li and received my PhD. from the Department of Civil Engineering, Tsinghua University in 2013. From 2011 to 2015, I visited the California Partners for Advanced Transit and Highways (PATH) Program, University of California at Berkeley, and worked at the University of Maryland, College Park, as a research associate and research director of the National Transportation Center. Since 2015, I have been working with the College of Civil Engineering and Architecture, Zhejiang University, Hangzhou, China, and was promoted to tenured professor in 2021. During 2022–2023, I served as vice dean of Zhejiang University-University of Illinois Urbana-Champaign Institute. Since 2022, I directed the Institute of Intelligent Transportation Systems at Zhejiang University, China. Empowered by data science and artificial intelligence, my research aims to make human mobility systems more efficient, intelligent, and sustainable.

**Ziyou Gao:** In 1994, I received my PhD in operations research and cybernetics from the Institute of Applied Mathematics, Chinese Academy of Sciences, China, and then worked at Beijing Jiaotong University. Currently, I am a professor at Beijing Jiaotong University, and a recipient of support from the National Natural Science Foundation of China for Distinguished Young Scholars. Furthermore, I serve as the chairman of the Society of Management Science and Engineering, foreign academician of the Russian Academy of Natural Sciences, and IET fellow. Over the past few decades, I have conducted systematic research in transportation and logistics management, complex systems modeling, and transportation system complexity. I am particularly interested in quantifying and predicting the role of human factors in various complex systems, which is also the problem we aim to solve in our recent work published in *Patterns*.^1^ I believe this is crucial to many fields, such as urban planning, infectious disease prevention and control, and public safety management.

### What motivated you to become a researcher? What is the fun part of being a researcher? Ii there anyone or anything that helped guide you on your path?

**YC**: My first exposure to academic research occurred during my sophomore year of undergraduate studies when I joined the research group of Professor Shuai Zhang, my master’s advisor. My initial academic role entailed assisting master’s students in developing code for numerical experiments. I vividly remember that the first experiment was to use C# language to implement teaching and learning algorithms to solve manufacturing services’ combination and optimization problems. Over more than three months of coding and experimentation, the moment I achieved satisfactory experimental performance and submitted the paper, I was instantly captivated by the allure and joy of scientific research. From then on, I decided to pursue my master’s and PhD studies and embark on a path toward becoming a researcher.

I think the fun of being a researcher lies in optimizing various decision-making problems, striving to improve performance, and pushing the boundaries of current knowledge. In my academic career, I appreciate my master’s supervisor for providing me with solid academic training. I would also like to thank my PhD supervisor, Professor Xiqun (Michael) Chen, for broadening my academic perspective, and enabling me to explore critical scientific questions at the forefront of the field.

**XC**: When I was a PhD student in 2008, the freeway Performance Measurement System (PeMS) motivated me to become a data researcher. Led by Professor Pravin Varaiy, PeMS was initiated as a PATH research project at the University of California, Berkeley. The original intent was to support the data needs of traffic engineering researchers. Researchers use PeMS’s database to analyze traffic behavior on a large scale. During 2012–2015, when I worked with Professor Lei Zhang at the University of Maryland, College Park, I was involved in several research projects, the support data of which were collected from the Regional Integrated Transportation Information System (RITIS). It is an automated data sharing, dissemination, and archiving system with many performance measures, dashboards, and visual analytics tools that help agencies gain situational awareness, measure performance, and communicate information between agencies and the public. After joining the College of Civil Engineering and Architecture, Zhejiang University, Hangzhou, China, I established the Transportation Data & Simulation Optimization Laboratory (TDSO Lab) in 2015. Also, I teach two courses related to data science, i.e., Transportation Big Data Analytics and Transportation Data Science. The fun part of artificial intelligence for transportation includes explaining the field applications of big data analytics methodologies through a series of case studies, such as spatio-temporal distribution of geolocation, human mobility patterns, shared mobility implementations, traffic congestion identification and characterization using fused data, travel demand evolution both on individual and population levels, and traffic incident analysis.

### What is the major research gap in modeling human mobility within urban agglomerations?

**XC and ZG:** In the past, limited transportation and road facilities often constrained people to stay within their city. With the rapid urbanization and continuous expansion of transportation networks, the connections between neighboring cities have grown increasingly tighter. It has given rise to urban agglomerations, fostering frequent movement and interaction among communities in various cities. Nonetheless, traditional research has primarily focused on analyzing travel mobility at a single scale, either examining human mobility patterns within a city or studying aggregated travel characteristics between cities. As the spatial scale increases, the complexity of human travel choices and the computational dimension of the model increase significantly. The efficient and accurate characterization of human movement between neighborhoods in different cities remains an open and challenging research question.

### What are the main contributions of your current research?

**YC and ZG:** Given that people’s mental representation of physical space exhibits a clear hierarchical structure, we endeavored to integrate advanced neural-based deep learning algorithms to model the human travel choice process as a cascaded multi-class classification problem, thereby replicating universal-scale laws of human mobility across various spatial scales. Compared to directly modeling travel patterns between communities within all cities, our proposed “decomposition-integration” modeling strategy predicts the movement probabilities between regions at different scales in a cascade manner and uses probabilistic constraints to hierarchically compress the solution space to improve mobility modeling accuracy. Also, we constructed novel deep learning frameworks, including cascaded convolutional networks and graph generative adversarial networks, to enhance the capture of complex and nonlinear mobility features.

### Who thought first to call it MSUM, were there other name ideas, and how do you envision MSUM contributing to your community?

**YC:** The term multi-scale unified model (MSUM) was my initial choice, but I had only defined it in the article^1^ as encompassing multiple spatial scales at the spatial level. Professor Chen posed the question of whether our model could simultaneously capture the mobility patterns of individuals and populations. As it turned out, multi-scale holds another dimension of meaning: the unification of the scales of the research object. Consequently, we provide a new research perspective for multi-scale unified human mobility modeling, and I believe our research can promote further development.

**XC and ZG**: Indeed, MSUM not only achieves unified modeling across multiple spatial scales but also captures the movement patterns of individuals and populations simultaneously. Compared to traditional mobility prediction, we simulate people’s daily hierarchical travel choice process and construct a cascaded deep neural network, which effectively reduces the choice solution space of travel destinations, thereby improving the accuracy of mobility prediction. We believe this decomposition-integration modeling strategy stands out as one of the key highlights of our study and can serve as a valuable reference for the community.

### Why is understanding human mobility patterns important and how will your model be applied in the future, in the areas of intelligent vehicles? Urban planning? Smart city?

**XC**: Urban agglomerations are complex and giant systems, where intricate travel flows are a macro-aggregation phenomenon caused by the choice behaviors of a vast number of micro-individual travelers. Consequently, in light of the rapid development and popularization of technologies such as smart cities and autonomous driving, exploring the complexity of travel behavior will become a core issue in future transportation systems management for smart cities. Taking autonomous vehicles as an example, our model can furnish insights into people’s travel preferences and the likelihood of their movements within urban clusters. This wealth of data can be seamlessly integrated into autonomous vehicle systems, substantially enhancing their predictive capabilities and responsiveness to human behaviors, ultimately resulting in heightened road safety. In addition, our model can also provide data support for identifying traffic bottlenecks, facilitating dynamic traffic management. Finally, it can effectively alleviate traffic congestion and improve overall urban mobility.

**ZG**: Within the intricate fabric of a transportation network, various components such as people, vehicles, and roads coalesce, engaging in coordinated interactions. Among these elements, people are behavioral participants and demand formulators. People’s needs, behaviors, and decisions significantly impact the functionality and efficiency of urban systems. Consequently, understanding people’s mobility patterns is crucial in effective urban governance and transportation management. For instance, in urban planning, a comprehensive grasp of human mobility patterns empowers city administrators to improve road layouts, optimize public transportation systems, and refine urban infrastructure, all tailored to align with the diverse travel needs of residents. This, in turn, engenders more sustainable, streamlined, and compassionate cities.

### How do you keep up to date with advances in both data science techniques and in your field? How can data science help your domain, and how can collaborations between academia and your domain be started?

**YC**: I keep updated with advances in the field by dedicating at least two hours each day to reading the latest papers. At the same time, I’ve honed my focus on several researchers whose work closely aligns with my research direction. Once they release a new paper, I will receive an e-mail notification. Additionally, I actively participate in various academic conferences, fostering in-person interactions and knowledge exchange with peers within the academic community.

In the fields of social science and transportation science, in-depth data analysis and mining provide decision-making support for managers. Relying solely on domain experts’ knowledge is no longer enough to resolve large-scale optimization challenges. The integration of data science into domain-specific intelligent decision-making processes offers a substantial enhancement in decision-making efficiency and solution precision.

**XC**: I stay abreast of developments in data science and related research frontiers by organizing international conferences and active participation in academic forums. For example, our team has hosted events, e.g., the Digital and Intelligent Transportation Forum, where we have invited esteemed experts and scholars in the field to share their latest research progress. These conferences serve as valuable platforms for me to gain insights into cutting-edge research trends and draw inspiration for my scientific endeavors.

In addition, a significant portion of our academic research is rooted in addressing real-world challenges. For instance, we developed a data-driven multi-agent-based modeling and simulation system software to provide a robust simulation evaluation of online ride-hailing and cruising vehicles under various supply scenarios. It is founded on a deep learning cloud control deployment architecture and is pivotal in facilitating data-driven decision-making in transportation management within Ningbo, China. Furthermore, we have proposed a spatio-temporal travel chain reconstruction method encompassing travel destinations, modes, and purposes. It furnishes algorithmic support for the ongoing advancement of City Brain’s travel platform in Hangzhou, China. In turn, the data generated by these platforms can also be harnessed to enhance our models’ training and refine our algorithmic strategies, creating a virtuous cycle of improvement and optimization.

### Which of the current trends in data science seem most interesting to you? In your opinion, what are the most pressing questions for the data science community?

**XC**: Data science and decision intelligence have become cutting-edge research directions. In recent years, top journals have successively unveiled groundbreaking findings based on large-scale experiments and artificial intelligence decision-making mechanisms. In comparison, traditional methods have limited applicability in ultra-large-scale networks and travel decisions of tens of millions of agents. It is necessary to promote the construction of smart cities with the guidance of digital twin cities, propose a cognitive intelligence method for the characteristics of information asymmetry, personalized decision-making, and dynamic games in the big data environment; propose a decision-making intelligence method for smart city transportation system function self-encapsulation, structural adaptation, and learning self-evolution; and build a multi-resolution digital twin model for multi-mode shared travel in smart cities, ultimately realizing scenario reproduction, simulation deduction, scenario derivation and generalization based on twin data, and provide strong support for digital governance of smart city traffic and smart travel.

In addition, one of the most pressing questions for the data science community is acquiring high-quality data. The reliability and accuracy of data are paramount for informed decision-making and meaningful analysis. Data scientists grapple with challenges related to data quality, especially when dealing with diverse sources and formats. Ensuring data integrity, addressing issues of bias, and reconciling discrepancies are essential tasks.

**YC**: One of the most intriguing trends in data science revolves around incorporating large-scale models, particularly in human mobility modeling. Leveraging massive pre-trained language models like GPT-4 for understanding and predicting human mobility patterns is an exciting frontier. These models, initially conceived for natural language tasks, hold the potential to capture the intricate behaviors and decision-making processes that underlie human movement in urban environments.

However, while implementing large models, safeguarding individuals’ sensitive information while extracting meaningful insights remains a pressing concern. With the growing importance of data-driven decision-making, it’s imperative that we develop robust approaches to anonymize and protect personal data. Striking the right balance between data utility and privacy preservation is a complex and ongoing challenge.

### Was there a particular element (paper, collaboration, talk/conference, key experiment, idea, result) that motivated you to start/participate in this project?

**YC**: In Professor Ziyou Gao’s previous article^2^ published in *Nature Communications*, they pioneered unified modeling for individual and population mobility. The model effectively captures multiple scaling laws governing human travel with a single adjustable parameter. It sparked my interest in scale-unified mobility modeling. As urbanization continues to accelerate, we have found that developing urban agglomerations is pivotal for a country’s sustainable regional growth. Modeling human mobility within urban agglomerations involves multiple spatial scales, which is more challenging than analyzing mobility patterns within or between cities. In 2021, a mobility prediction model^3^ based on deep neural networks was published in *Nature Communications*, demonstrating the powerful advantages of deep learning algorithms in understanding and predicting human mobility. Drawing inspiration from these pivotal developments, we proposed an ingenious deep learning framework to enhance the precision of human mobility prediction across multiple spatial scales within urban agglomerations, leading to this project’s inception.

### What drew you to this area of research? How has the research focus of your team evolved over the years?

**XC**: Big data mining and analysis of urban travel is a current research hotspot. By cooperating with mobile operators and conducting data analysis on anonymous mobile phone signaling, we can capture the distribution of people’s residences, workplaces, and population density at different times, which then allows us to understand the travel characteristics of residents. Through microwave data and radar data, we can understand the driving conditions of motor vehicles. Regarding public transportation, we also collect public transit automated fare collection (AFC) data. Building upon this wealth of city travel-related big data, I established the TDSO Lab at Zhejiang University. The core research direction of our laboratory can be summarized by three keywords: big data analytics, transportation simulation, and system optimization. Furthermore, in response to the growing prominence of shared mobility and the goal of maximizing resource sharing and utilization, we have delved deeply into studying the behavioral mechanisms and various issues surrounding shared mobility through data modeling. Over the past few years, our team has conducted continuous and thorough research in this area, yielding fruitful results.

### How did you introduce your team to the community? Were conferences important in this? What support did you get from the community?

**XC**: I usually introduce our team by holding conferences, publishing research results, and cooperating with relevant companies or institutions. Conferences (e.g., the *IEEE International Conference on Intelligent Transportation Systems*, the *World Transport Convention*, and the *International Workshop on Computational Transportation Science*) are an effective platform to communicate with fellow experts, academics, and industry, providing excellent opportunities to connect with the community and gain wider recognition for our work. It is also essential to cooperate with relevant organizations to jointly promote research and innovation. During the cooperation process, we can obtain data, financial, and other support from enterprises, governments, and other community sectors. At the same time, jointly publishing relevant products with industry partners (e.g., Alibaba, DiDi Chuxing, and Huawei) helps expand the academic influence of our team.

### How important was the collaboration to the success of the paper? How important do you think collaboration is in general to research?

**ZG**: Collaboration brought together individuals with diverse expertise and backgrounds. Each collaborator brought unique insights, skills, and perspectives to the collaboration, enriching the research process. This diversity of expertise allowed us to tackle complex problems and devise innovative research methodologies. For example, our research requires close collaboration between researchers with professional backgrounds in computer science, systems science, and transportation science. Researchers in systems science and transportation science solve problems in human mobility modeling and establish solutions to multi-scale mobility prediction. Computer scientists process and analyze data and tune deep neural networks. Interdisciplinary communication and cooperation ensured the output of high-quality results in this paper.

## References

[1] Chen Y., Xu H.G., Gao Z.Y., Chen X.C. (2023). A multi-scale unified model of human mobility in urban agglomerations. Patterns,.

[2] Yan X.Y., Wang W.X., Gao Z.Y., Lai Y.C. (2017). Universal model of individual and population mobility on diverse spatial scales. Nat. Commun..

[3] Simini F., Barlacchi G., Luca M., Pappalardo L. (2021). A deep gravity model for mobility flows generation. Nat. Commun..

